# Live combined bifidobacterium attenuates rivaroxaban-associated colonic bleeding in mice: evidence for a substantial gut microbiota-dependent component

**DOI:** 10.3389/fphar.2026.1846463

**Published:** 2026-10-21

**Authors:** Xiaowen Chen, Yuze Lin, Youqi Huang, Linghong Zeng, Jie Lin, Min Chen

**Affiliations:** 1 Shengli Clinical College of Fujian Medical University, Department of Pharmacy, Fujian Provincial Hospital, Fuzhou University Affiliated Provincial Hospital, Fuzhou, China; 2 Shengli Clinical College of Fujian Medical University, Department of Pharmacy, College of Pharmacy, Fujian Medical University, Fuzhou University Affiliated Provincial Hospital, Fuzhou, China; 3 Longyan City Center for Drug Inspection and Testing, Longyan, China; 4 Department of Pathology, Shengli Clinical Medical College of Fujian Medical University, Fujian Provincial Hospital, Fuzhou University Affiliated Provincial Hospital, Fuzhou, Fujian, China

**Keywords:** 16S rRNA gene sequencing, colonic bleeding, gut microbiota, live combined bifidobacterium, rivaroxaban, untargeted metabolomics

## Abstract

Rivaroxaban, a direct oral anticoagulant (DOAC), is widely prescribed for the prevention and treatment of thromboembolic disorders. However, its use is associated with an increased risk of gastrointestinal bleeding, the mechanisms of which remain incompletely understood. Effective strategies to relief such bleeding are also lacking. Because the gut microbiota plays a critical role in maintaining intestinal homeostasis, we investigated whether probiotics could mitigate anticoagulant-related bleeding and explored the underlying mechanisms. Using a rivaroxaban-induced mouse model of colonic bleeding, we found that treatment with live combined Bifidobacterium reduced occult blood in the feces, alleviated damage to colon tissue, and lowered serum levels of the inflammatory cytokines tumor necrosis factor-α and chemokine ligand 5. Integrated 16S rRNA sequencing and untargeted metabolomic analyses showed that LCB treatment was accompanied by alterations in the gut microbial community and fecal metabolic profile. Following antibiotic-mediated microbiota depletion, the overall ameliorative effect of LCB was attenuated, although partial responses, including a reduction in CXCL5, were retained. These findings support a substantial microbiota-dependent component of the full ameliorative phenotype. Untargeted metabolomic analysis identified a metabolite feature putatively annotated as anandamide that showed a nominal decrease following rivaroxaban administration and a nominal increase after LCB treatment. However, these differences did not remain significant after false discovery rate (FDR) correction and were therefore considered exploratory. The same feature also showed a nominal increase following LCB treatment in antibiotic-treated mice without corresponding restoration of the overall ameliorative phenotype, suggesting that this metabolic change alone was insufficient to account for the observed effects. To our knowledge, the present study provides initial evidence that LCB administered after the onset of fecal occult bleeding ameliorates rivaroxaban-associated colonic injury in mice. Collectively, these findings suggest that the overall ameliorative effect of LCB has a substantial gut microbiota-dependent component, whereas the putative anandamide signal represents a hypothesis-generating metabolic association that requires targeted quantitative and functional validation.

## Introduction

Rivaroxaban, a direct oral anticoagulant (DOAC), functions by specifically inhibiting coagulation factor Xa. Because it is taken orally and does not require routine monitoring of the international normalized ratio (INR), it has been widely adopted for the prevention and treatment of thromboembolic disorders. Multiple clinical studies have shown that, compared to warfarin, rivaroxaban carries a significantly higher risk of gastrointestinal bleeding (Chang et al., 2015; Loffredo et al., 2015; Gu et al., 2020). In addition, a recent analysis of the Food and Drug Administration Adverse Event Reporting System (FAERS) identified “gastrointestinal haemorrhage,” “rectal hemorrhage,” and “upper gastrointestinal hemorrhage” as the most frequently reported adverse events associated with this drug (Wu et al., 2024).

The mechanisms underlying rivaroxaban-induced gastrointestinal bleeding remain incompletely understood. While rivaroxaban produces systemic anticoagulation through factor Xa inhibition, its active form is excreted into the intestinal tract, where it may directly irritate vulnerable mucosa and thereby increase bleeding risk (Saviano et al., 2022). Current management strategies for rivaroxaban-associated gastrointestinal bleeding, such as discontinuing the drug, administering hemostatic agents, and/or performing interventional procedures, are reactive rather than preventive. Moreover, interrupting rivaroxaban therapy can compromise anticoagulation for the underlying condition. Thus, identifying strategies that reduce gastrointestinal bleeding without diminishing the efficacy of rivaroxaban is of significant clinical importance.

The gut microbiota, a complex community of microorganisms in the intestinal tract, plays a central role in maintaining the integrity of the intestinal barrier, regulating immune homeostasis, and metabolizing host-derived substances (de Vos et al., 2022). Interactions between the microbiota and the host influence not only the intestinal environment but also systemic health through effects on endocrine function, energy metabolism, and immune responses (Pickard et al., 2017; Adak and Khan, 2019). Probiotics, as microecological regulators, benefit intestinal health by excluding pathogens, strengthening barrier function, modulating immunity, and producing bioactive metabolites (Rose et al., 2021). Animal studies have shown, for example, that *Lactobacillus casei* Shirota can attenuate indomethacin-induced injury to the small intestine (Watanabe et al., 2009). Clinical evidence also suggests that the composition of the gut microbiota may influence bleeding risk in patients with atrial fibrillation receiving anticoagulant therapy (Li et al., 2023). Therefore, modulation of the gut microbiota with probiotics may represent a potential adjunctive approach for ameliorating anticoagulant-associated gastrointestinal bleeding.

A multi-strain probiotic preparation marketed as “live combined Bifidobacterium” (LCB) contains *Bifidobacterium longum* subsp. *infantis*, *Lactobacillus acidophilus*, *Enterococcus faecalis*, and *Bacillus cereus* and is used to improve intestinal function (Bai et al., 2023; Luo et al., 2023). This formulation has been shown to help restore balance in the gut microbiota by supplementing beneficial bacteria and suppressing potentially harmful microorganisms (Bai et al., 2020). However, its potential value in protecting the gastrointestinal tract against anticoagulant-associated injury has not been established.

Rivaroxaban administration can also injure colon tissue in mice, leading to bleeding, suggesting that the colon may be a primary site of rivaroxaban-associated gastrointestinal bleeding. Therefore, in this study, we investigated, for the first time, the ameliorative effect of probiotics against rivaroxaban-induced bleeding in the colon. By focusing on the role of the gut microbiota and its metabolites, we aimed to clarify the mechanisms through which LCB alleviates rivaroxaban-related hemorrhage in the colon. Our findings may provide an experimental basis for developing therapeutic strategies against DOAC-induced gastrointestinal bleeding in clinical practice.

## Materials and methods

### Chemicals and reagents

Rivaroxaban (purity ≥98%, CAS: 366,789–02–8), phosphate-buffered saline (PBS), dimethyl sulfoxide (DMSO, CAS: 67–68–5), vancomycin (purity ≥98%, CAS: 1404–90–6), neomycin (potency ≥600 μg/mg, CAS: 1405–10–3), metronidazole (purity ≥99%, CAS: 443–48–1), and ampicillin (purity ≥99%, CAS: 7177–48–2) were purchased from Pusitang Biotechnology Co., Ltd. (Beijing, China). HPLC-grade methanol, acetonitrile, formic acid, water, and isopropanol were obtained from Thermo Fisher Scientific (Shanghai, China).

### Probiotics

LCB (*Bifidobacterium longum* subsp. *infantis* CGMCC0460.1, *Lactobacillus acidophilus* CGMCC0460.2, *Enterococcus faecalis* CGMCC0460.3, and *Bacillus cereus* CGMCC0460.4) was obtained from Hangzhou Grand Biopharmaceutical Co., Ltd. (Hangzhou, China). For the experiments, probiotic powder was dissolved in PBS to prepare a mixed suspension at the following concentrations: *B. longum subspecies infantis* (9.375 × 10^4^ CFU/mL), *L. acidophilus* (9.375 × 10^4^ CFU/mL), *E. faecalis* (9.375 × 10^4^ CFU/mL), and *B. cereus* (9.375 × 10^3^ CFU/mL). The suspension was administered once daily by oral gavage at 1 μL/g body weight (20 μL for a 20-g mouse), equivalent to 31.25 mg/kg/day. For a 20-g mouse, this corresponded to a minimum nominal total probiotic dose of approximately 5.81 × 10^3 CFU/mouse/day.

### Animals

Thirty 7-week-old specific pathogen-free (SPF) male C57BL/6J mice (20 ± 2 g) were used in the two main experiments described below. In addition, an independent preliminary cohort of 10 mice, examined before the main experiments, was used to assess the effect of the antibiotic regimen on the gut microbial community. All mice were obtained from Shanghai SLAC Laboratory Animal Co., Ltd. (Shanghai, China). The animals were housed under a 12 h light/dark cycle at an ambient temperature of 21 °C ± 2 °C and relative humidity of 50% ± 10%, with free access to food and water. All mice were allowed to acclimate for 7 days prior to the initiation of the experiments. All animal procedures were approved by the Ethics Committee of Fujian Provincial Hospital, Fuzhou University Affiliated (Approval No. IACUC-FPH-SL-20250117 [0488]).

Experiment 1: Eighteen mice were randomly divided into three groups (n = 6 per group): normal control (NC), rivaroxaban model (R), and LCB treatment (RB). Rivaroxaban was dissolved in PBS with the aid of dimethyl sulfoxide (DMSO) and ultrasonication to ensure complete solubilization. The final concentration of dimethyl sulfoxide (DMSO) in the rivaroxaban gavage solution was less than 0.1% (v/v), and both the R and RB groups received identical vehicle containing rivaroxaban. The NC group received PBS without DMSO. Mice in the R and RB groups received rivaroxaban (9 mg/kg) by oral gavage once daily for 7 consecutive days. Subsequently, the R group received PBS by oral gavage for 7 days, whereas the RB group received LCB once daily by oral gavage at the body-weight-adjusted dose described above for 7 days. The NC group received PBS by oral gavage for 14 consecutive days.

Experiment 2: Twelve mice were randomly divided into two groups (n = 6 per group): antibiotic-treated + rivaroxaban (ABX-R) and antibiotic-treated + rivaroxaban + LCB treatment (ABX-RB). To establish the antibiotic-treated model, all mice received a daily oral gavage of an antibiotic cocktail (vancomycin 50 mg/kg, neomycin 100 mg/kg, metronidazole 100 mg/kg) for 7 consecutive days to induce substantial antibiotic-mediated disruption of the gut microbiota. Concurrently, ampicillin was dissolved in sterile PBS at 1 mg/mL and supplied to the mice in drinking water for the duration of antibiotic treatment. Following antibiotic pretreatment, both groups were administered rivaroxaban (9 mg/kg) by oral gavage once daily for 7 consecutive days (days 8–14). Subsequently, the ABX-RB group received LCB once daily by oral gavage at the body-weight-adjusted dose described above for 7 days (days 15–21). During this period, the ABX-R group received sterile PBS by oral gavage for 7 consecutive days.

Independent preliminary experiment: An additional cohort of 10 mice was examined in a preliminary experiment conducted before the two main experiments. This cohort comprised a control group (n = 5) and an antibiotic-treated group (n = 5). The antibiotic-treated mice received the same 7-day antibiotic regimen subsequently used in Experiment 2. Fecal samples were collected after completion of the 7-day antibiotic treatment and analyzed by 16S rRNA gene sequencing. These mice were independent of the animals used in Experiments 1 and 2, and the corresponding results are presented in Supplementary Figure S1.

Blood samples were collected from the retro-orbital sinus, and serum was obtained via centrifugation at 3000 rpm for 15 min at 4 °C. Following blood collection, the mice were euthanized by intraperitoneal injection of sodium pentobarbital (150 mg/kg). Colon tissues were promptly excised and rinsed with saline. Both colon tissue samples and serum were immediately stored at −80 °C for subsequent analysis.

### Fecal occult blood test

Occult blood in fresh fecal samples was assessed daily using a commercial fecal occult blood test kit based on the pyramidon method (Leagene Biotechnology Co., Ltd., Beijing, China) according to the manufacturer’s instructions. Briefly, approximately 10–50 mg of freshly collected feces was tested, and color development was evaluated within 2 min after addition of the reagents. The manufacturer’s five ordinal reaction categories were numerically coded as follows: 0, negative, defined as no color development within 2 min; 1, weak positive (+), defined as a gradual change from colorless to light purple or purple after 10 s; 2, moderate positive (2+), defined as an initially light-purple reaction that gradually developed into distinct purple; 3, strong positive (3+), defined as an immediate purple reaction that progressively became dark purple; and 4, very strong positive (4+), defined as an immediate dark-purple reaction. Higher scores therefore represented a more rapid and intense color reaction and a greater semiquantitative fecal occult blood signal.

### Hematoxylin and eosin (H&E) staining

H&E staining was performed to evaluate histopathological changes in colon tissue. Colon segments (5 mm in length) were collected, fixed, embedded in paraffin, and sectioned at a thickness of 5 μm. Then they were stained with hematoxylin and eosin for histological examination.

Histopathological assessment was performed by a pathologist blinded to treatment allocation. Histopathological injury was assessed using a predefined semiquantitative scoring system comprising four domains: inflammatory cell infiltration, crypt and epithelial injury, vascular congestion and tissue edema, and intratissue hemorrhage. Each domain was scored from 0 to 3, yielding a total histopathological injury score ranging from 0 to 12, with higher scores indicating more severe colonic injury. The detailed scoring criteria are provided in Table 1.

**TABLE 1:** Histopathological scoring criteria for rivaroxaban-associated colonic injury (total score: 0–12).

Histopathological parameter	0 score	1 score	2 scores	3 scores
Inflammatory cell infiltration	No apparent inflammatory cell infiltration	Mild, scattered inflammatory cell infiltration confined to the mucosa	Moderate, multifocal inflammatory cell infiltration involving the mucosa and submucosa	Dense and extensive inflammatory cell infiltration involving multiple tissue layers or forming prominent inflammatory foci
Crypt and epithelial injury	Intact epithelium with regularly arranged crypts	Mild epithelial swelling or focal disorganization of crypt architecture	Partial crypt loss or deformation, with focal epithelial detachment	Extensive crypt destruction accompanied by mucosal erosion or ulceration
Vascular congestion and tissue edema	No vascular congestion or tissue edema	Mild capillary dilation with focal congestion	Marked vascular dilation and diffuse congestion accompanied by mild-to-moderate tissue edema	Severe vascular dilation and congestion accompanied by marked submucosal edema
Intratissue hemorrhage	No erythrocyte extravasation	Mild, focal erythrocyte extravasation	Multifocal erythrocyte extravasation or small patchy hemorrhagic areas	Extensive patchy hemorrhage, hematoma-like changes, or marked erythrocyte accumulation

### Enzyme-linked immunosorbent assay (ELISA)

Serum levels of inflammatory cytokines, including tumor necrosis factor-alpha (TNF-α) and chemokine ligand 5 (CXCL5), were measured using a commercial ELISA kit (Servicebio Technology Co., Ltd., Wuhan, China) according to the manufacturer’s instructions.

### 16S rRNA gene sequencing

Fecal samples were collected in sterile centrifuge tubes and immediately stored at −80 °C. Genomic DNA was extracted from fecal samples using the MagPure Soil DNA LQ Kit (Tiangen Biotech Co., Ltd., Beijing, China) according to the manufacturer’s instructions. DNA concentration and purity were assessed using a NanoDrop 2000 spectrophotometer (Thermo Fisher Scientific, USA) and agarose gel electrophoresis. The extracted DNA was stored at −20 °C until further analysis.

PCR amplification of the bacterial 16S rRNA gene was performed using extracted genomic DNA as the template, Takara Ex Taq high-fidelity polymerase, and barcoded primers. The V3-V4 hypervariable region of the 16S rRNA gene was amplified using the universal primers 343F (5′-TACGGRAGGCAGCAG-3′) and 798R (5′-AGG​GTA​TCT​AAT​CCT-3′) to analyze bacterial diversity (Nossa et al., 2010). PCR products were examined via agarose gel electrophoresis. Following purification, sequencing was performed on the Illumina NovaSeq 6000 platform (Illumina, San Diego, CA, USA) to generate 250 bp paired-end reads.

For both Experiment 1 and Experiment 2, four mice per group were randomly selected from the total of six mice per group for 16S rRNA sequencing and metabolomic analyses. No animals were excluded from any analysis. The selection was made prior to data acquisition and was independent of phenotypic outcomes.

For the independent preliminary cohort, fecal samples from all five control and five antibiotic-treated mice were subjected to 16S rRNA gene sequencing using the same procedures described above.

### Bioinformatics analysis

Downstream microbiome diversity analyses and the corresponding visualizations were performed using R software version 3.3.1. Alpha diversity was assessed using the Chao1, Shannon, and Simpson indices, as applicable. Chao1 was interpreted as an estimator of community richness, whereas the Shannon and Simpson indices were used to characterize alpha diversity incorporating information on richness and evenness. For Experiment 1, overall differences in the Shannon and Simpson indices among the NC, R, and RB groups were evaluated using Kruskal–Wallis rank-sum tests, followed by Dunn’s post-hoc pairwise comparisons, with false discovery rate (FDR) correction for multiple testing. For Experiment 2, Shannon and Simpson indices were compared between the ABX-R and ABX-RB groups using two-sided Wilcoxon rank-sum tests. In the independent preliminary antibiotic-validation cohort, Chao1 and Shannon indices were compared using two-sided Wilcoxon rank-sum tests.

Beta diversity was assessed using principal coordinate analysis (PCoA) based on Bray–Curtis dissimilarities. Differences in overall microbial community structure were evaluated using analysis of similarities (ANOSIM) in Experiments 1 and 2. For the independent preliminary antibiotic-validation cohort, differences in community structure were assessed using permutational multivariate analysis of variance (PERMANOVA) based on Bray–Curtis dissimilarities.

Linear discriminant analysis effect size (LEfSe) was used to identify discriminative bacterial taxa, with a significance criterion of P < 0.05 and an LDA score threshold >2.0. For the three-group comparison in Experiment 1, the all-against-all strategy was used.

### Metabolomics analysis

Metabolite extraction was performed according to sample type. For solid samples (feces), cryogenic grinding was used, followed by extraction with a methanol-water solution (methanol:water = 4:1, v/v) containing four internal standards (including 0.02 mg/mL L-2-chlorophenylalanine). The mixture was subjected to low-temperature ultrasonication, allowed to stand, and then centrifuged. The supernatant was collected for subsequent analysis. QC samples were prepared by pooling equal volumes of each sample; these were regularly injected throughout the analytical sequence to monitor instrument stability and data reproducibility.

Liquid chromatography-mass spectrometry (LC-MS) analysis was performed on a Thermo Fisher Scientific (Waltham, MA, USA) UHPLC-Q Exactive system (ultra-high-performance liquid chromatography coupled with Fourier-transform mass spectrometry). Separation was achieved using an HSS T3 column (100 mm × 2.1 mm, 1.8 μm, Waters, Milford, MA, USA). The mobile phase consisted of solvent A (water/acetonitrile [95/5, v/v] with 0.1% formic acid) and solvent B (acetonitrile/isopropanol/water [47.5/47.5/5, v/v/v] with 0.1% formic acid). MS data were acquired in both positive and negative ion scanning modes over a mass-to-charge ratio (m/z) range of 70–1050. High-resolution MS provided accurate precursor mass and fragment information for metabolite identification.

Principal component analysis (PCA) was subsequently performed to assess global metabolic differences between groups. After normalization and log transformation, the metabolomic data were Pareto-scaled prior to supervised multivariate analysis. OPLS-DA models were constructed using the ropls package (version 1.6.2) in R and evaluated using seven-fold cross-validation. Model performance was summarized using R^2^X, R^2^Y, and Q^2^, and potential overfitting was further assessed using 200 permutation tests with randomized class labels. For univariate analysis, nominal P values for metabolite features in the NC *versus* R, R *versus* RB, and ABX-R *versus* ABX-RB comparisons were calculated using two-sided unpaired Student’s t-tests. Formal feature-wise tests of normality and homogeneity of variance were not performed. Given the limited sample size (n = 4 per group), these assumptions could not be robustly evaluated; therefore, the nominal univariate results were interpreted as exploratory. Metabolite features were initially screened using a variable importance in projection (VIP) score >1 derived from the corresponding OPLS-DA model together with a nominal P value <0.05. To account for multiple testing, P values were additionally adjusted using the Benjamini–Hochberg false discovery rate (FDR) procedure separately within each pairwise comparison. Features with FDR-adjusted P < 0.05 were considered statistically supported after multiple-testing correction, whereas features meeting VIP >1 and nominal P < 0.05 but not the FDR threshold were considered exploratory candidate metabolite features. Both nominal and FDR-adjusted P values are provided in Supplementary Table S1. The same four randomly selected mice per group used for 16S rRNA sequencing were also used for untargeted metabolomic analysis.

### Statistical analysis

Phenotypic and histopathological analyses were performed using GraphPad Prism version 10.6.0 (GraphPad Software, Boston, MA, USA). Longitudinal body-weight data were analyzed using two-way repeated-measures ANOVA, with treatment group and time as factors. Sphericity was not assumed, and the Geisser-Greenhouse correction was applied. Where appropriate, between-group comparisons at individual time points were performed using Sidak’s multiple-comparisons test.

Because the fecal occult blood score was an ordinal semiquantitative outcome, it was analyzed separately from body weight. Between-group comparisons were performed separately at each daily time point using two-sided Mann–Whitney U tests. To control the family-wise error rate arising from repeated day-by-day testing, P values were adjusted using the Bonferroni method within each experiment. The 14 daily comparisons in Experiment 1 (days 1–14) and the 21 daily comparisons in Experiment 2 (days 1–21) were treated as separate families of comparisons. Because the corresponding groups received identical treatments before initiation of LCB intervention, comparisons during the pre-intervention periods were not interpreted as treatment effects of LCB. For Experiment 1, comparisons between the R and RB groups were performed across days 1–14, whereas for Experiment 2, comparisons between the ABX-R and ABX-RB groups were performed across days 1–21. Because the corresponding groups received identical treatments before initiation of LCB intervention, comparisons during the pre-intervention periods were not interpreted as treatment effects of LCB. Fecal occult blood scores are presented as median with interquartile range.

For single-time-point data, comparisons among three groups were performed using one-way ANOVA followed by Tukey’s multiple-comparisons test or the Kruskal–Wallis test followed by Dunn’s test, as appropriate. Comparisons between two groups were performed using an unpaired two-tailed Student’s t-test or the Mann-Whitney U test, as appropriate. Histopathological injury scores were analyzed using the Kruskal–Wallis test followed by Dunn’s test for three-group comparisons and the Mann-Whitney U test for two-group comparisons.

Exploratory associations between genus-level bacterial relative abundances and the normalized abundance of the metabolite feature putatively annotated as anandamide were evaluated using Spearman’s rank correlation analysis. Microbiome and metabolomic datasets were matched by individual animal identifiers, and only mice with paired measurements were included. The analysis comprised 20 mice from five experimental groups (NC, R, RB, ABX-R, and ABX-RB; n = 4 per group). Spearman’s correlation coefficients and corresponding two-sided asymptotic P values were calculated using the scipy. stats.spearmanr () function in Python version 2.7.10 with SciPy version 1.0.0. Associations were included in the exploratory correlation network when |ρ| > 0.4 and nominal P < 0.05. To account for multiple correlation testing, nominal P values were additionally adjusted using the Benjamini–Hochberg procedure across all tested correlations. Network inclusion was based on the predefined nominal P-value threshold, whereas BH-adjusted P values were reported to indicate statistical support after multiple-testing correction.

## Results

### LCB attenuates rivaroxaban-induced colonic bleeding in mice

The experimental design for establishing the rivaroxaban-induced colonic bleeding model and subsequent LCB intervention is illustrated in Figure 1A. Two-way repeated-measures ANOVA with Geisser–Greenhouse correction revealed a significant effect of time on body weight [F (6.275, 94.12) = 4.506, P = 0.0004], but no significant group effect [F (2, 15) = 2.500, P = 0.1156] or group × time interaction [F (12.55, 94.12) = 1.207, P = 0.2879] (Figure 1B). Fecal occult blood became detectable after rivaroxaban administration and progressively increased during the first 7 days in both the R and RB groups, during which both groups received the same rivaroxaban treatment. Following initiation of LCB treatment on day 8, fecal occult blood scores were generally lower in the RB group than in the R group. Day-by-day comparisons across the complete 14-day observation period were performed using two-sided Mann–Whitney U tests with Bonferroni adjustment for the 14 daily comparisons, and a significant between-group difference was observed on day 9 (adjusted P = 0.03) (Figure 1C). In addition, serum levels of TNF-α and CXCL5 were significantly elevated in the R group compared to the NC group. However, this increase was markedly attenuated following LCB treatment, suggesting that probiotic intervention mitigates the systemic inflammatory response associated with rivaroxaban administration (Figure 1D). Histopathological examination of colon tissues revealed capillary congestion and inflammatory cell infiltration in the R group. In contrast, these pathological changes were substantially ameliorated in mice that received the treatment (Figure 1E). Blinded semiquantitative histopathological scoring was performed to quantitatively evaluate colonic injury. The total injury score was significantly higher in the R group than in the NC group, whereas the RB group showed a significantly lower score than the R group (Figure 1F). No significant difference was observed between the NC and RB groups. These quantitative findings were consistent with the representative histological observations and supported the ameliorative effect of LCB on rivaroxaban-associated colonic injury. Taken together, these findings indicate that LCB administered after rivaroxaban exposure ameliorated the existing fecal bleeding phenotype, inflammatory response, and colonic histopathological alterations in mice.

**FIGURE 1:**
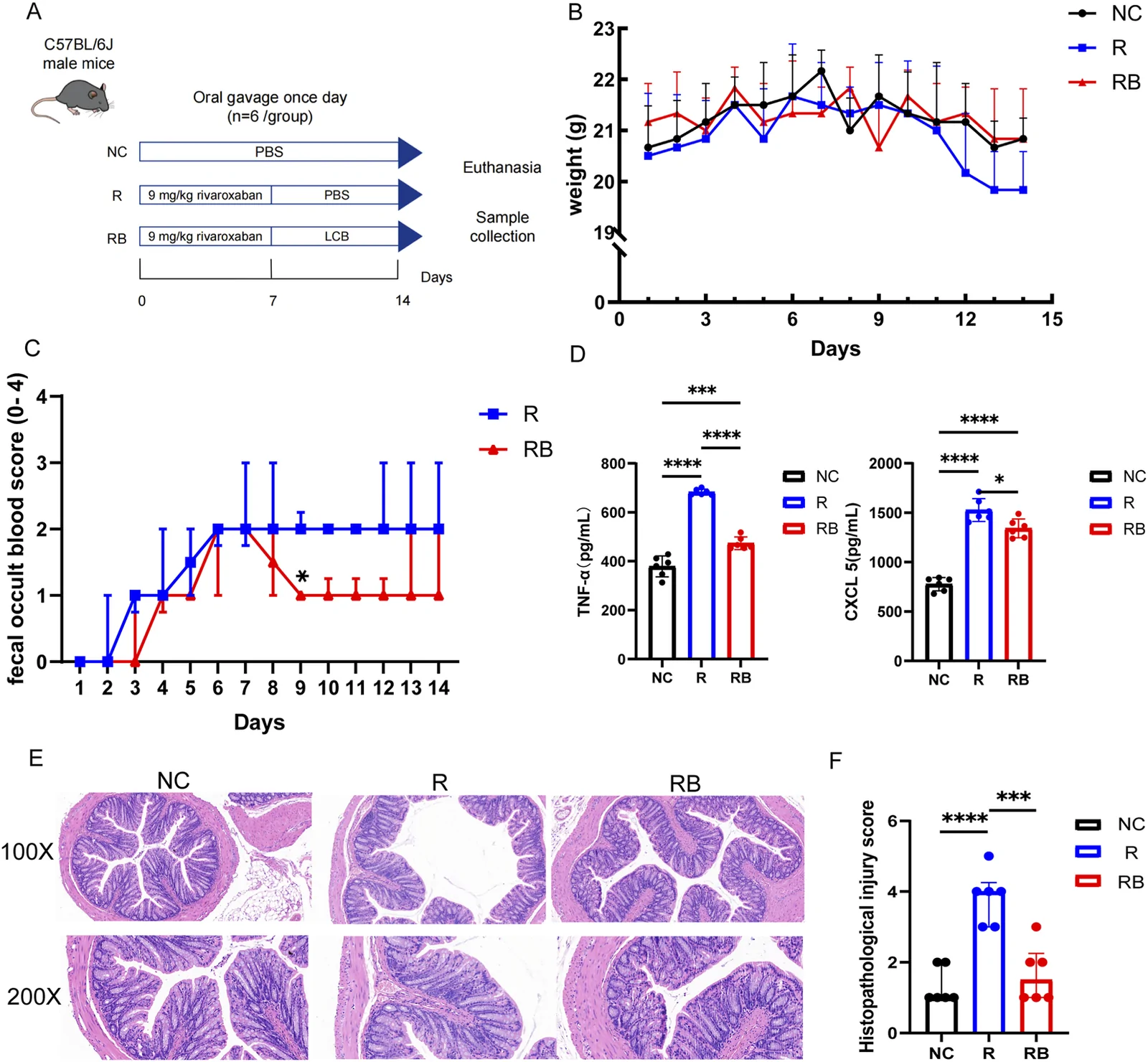
LCB alleviates rivaroxaban-induced colonic bleeding in mice. **(A)** Schematic diagram of the experimental protocol. **(B)** Changes in body weight in mice throughout the experimental period. **(C)** Levels of fecal occult blood in mice. **(D)** Serum levels of TNF-α and CXCL5. **(E)** Representative H&E-stained colon sections at 100× and 200× total magnification. Scale bars: 50 μm for the 100× images and 25 μm for the 200× images. **(F)** Blinded semiquantitative histopathological injury scores. Continuous data are presented as mean ± SD, whereas fecal occult blood scores and histopathological injury scores are presented as median with interquartile range (n = 6 per group). *P < 0.05, ***P < 0.001, ****P < 0.0001.

### LCB modulates the gut microbiota of mice with rivaroxaban-induced colonic bleeding

We further investigated whether the protective effect of LCB was associated with modulation of the gut microbiota. Alpha diversity was assessed using the Shannon and Simpson indices, which characterize community diversity by incorporating information on richness and evenness. Significant overall differences among the NC, R, and RB groups were observed for both the Shannon index (Kruskal–Wallis P = 0.0373, FDR-adjusted P = 0.0448) and the Simpson index (Kruskal–Wallis P = 0.0097, FDR-adjusted P = 0.0362) (Figure 2A). Dunn’s post-hoc analysis with FDR correction identified a significant pairwise difference in the Simpson index between the NC and R groups, whereas no significant pairwise differences were identified for the Shannon index. Beta diversity analysis using PCoA based on Bray–Curtis dissimilarities revealed clear separation of the gut microbial communities among the three experimental groups. ANOSIM confirmed a strong between-group difference in overall microbial community structure (R = 1.000, P = 0.010) (Figure 2B).

**FIGURE 2:**
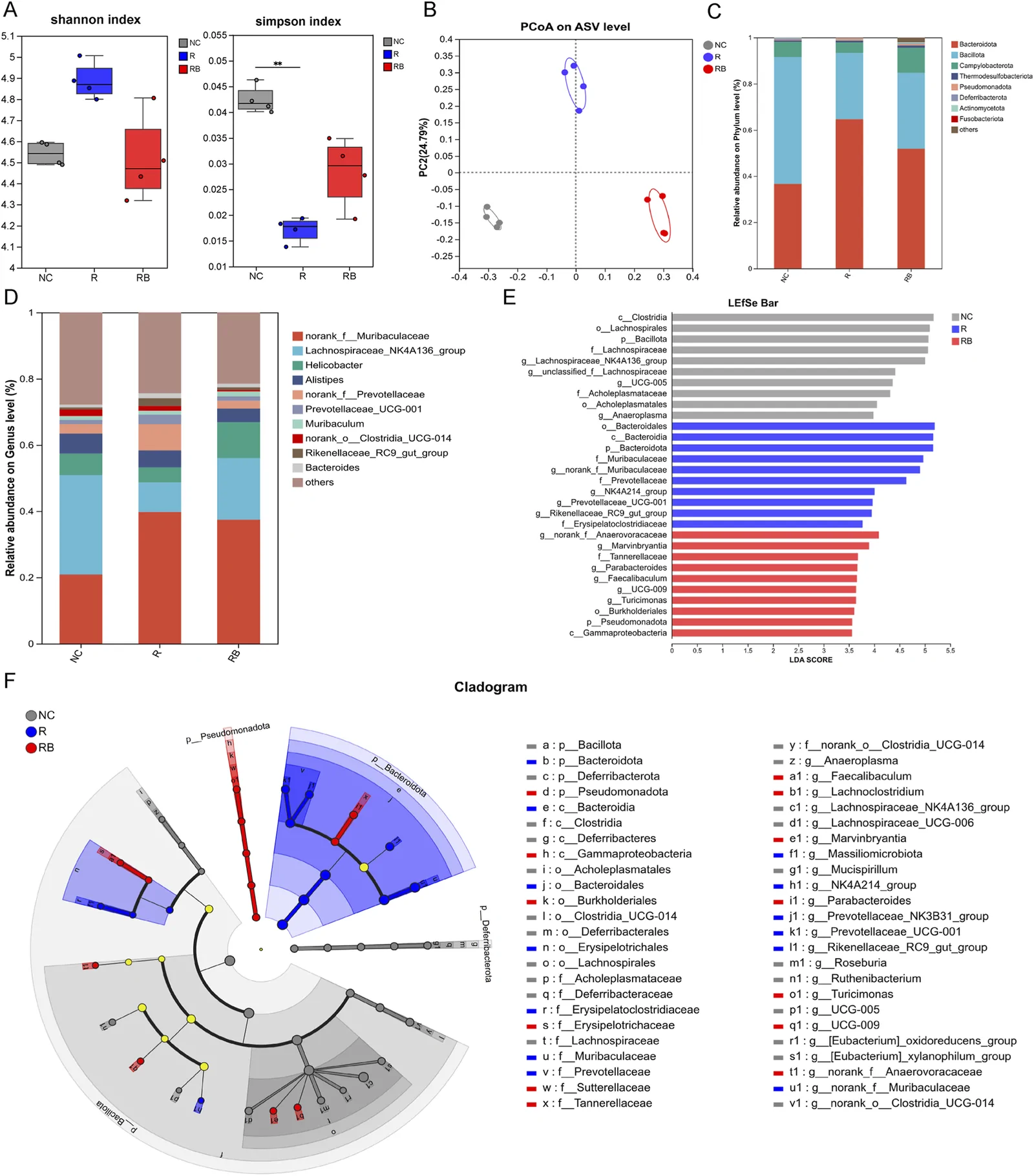
Effect of LCB intervention on the gut microbiota of mice with rivaroxaban-induced colonic bleeding. **(A)** Alpha diversity assessed using the Shannon and Simpson indices. Overall group differences were evaluated using Kruskal–Wallis rank-sum tests followed by Dunn’s post-hoc pairwise comparisons, with FDR correction for multiple testing. Significant pairwise comparisons identified by the FDR-corrected Dunn’s post-hoc analysis are indicated in figure. **(B)** PCoA based on Bray–Curtis dissimilarities; differences in overall community structure were evaluated using ANOSIM (R = 1.000, P = 0.010). **(C)** Gut microbial composition at the phylum level. **(D)** Gut microbial composition at the genus level. **(E)** LDA score plot showing discriminative taxa among the NC, R, and RB groups. **(F)** LEfSe cladogram showing the phylogenetic distribution of discriminative taxa. LEfSe features were identified using P < 0.05 and an LDA score >2.0, with the all-against-all strategy used for the three-group comparison. Data are based on n = 4 randomly selected mice per group for 16S rRNA sequencing.

To characterize the specific compositional changes induced by rivaroxaban and probiotic intervention, we analyzed the relative abundance of bacterial taxa at the phylum and genus levels. At the phylum level, rivaroxaban treatment increased the abundance of Bacteroidota while decreasing those of Bacillota and Campylobacterota compared to the NC group. These changes were reversed by LCB treatment (Figure 2C). At the genus level, the R group exhibited enrichment of norank_f_Muribaculaceae, norank_f_Prevotellaceae, Prevotellaceae_UCG-001, and Rikenellaceae_RC9_gut_group, accompanied by a reduction in Lachnospiraceae_NK4A136_group and *Helicobacter* relative to the NC group. LCB treatment reversed these trends, restoring the abundances of these taxa toward control levels (Figure 2D). LEfSe was performed to identify differentially abundant taxa among groups (Figures 2E,F). The NC group was characterized by enrichment of c*_Clostridia*, o*_Lachnospirales*, p*_Bacillota*, f*_Lachnospiraceae*, and g*_Lachnospiraceae_NK4A136_group*. In contrast, the R group showed significant enrichment of p*_Bacteroidota*, c*_Bacteroidia*, o*_Bacteroidales*, f*_Muribaculaceae*, and f*_Prevotellaceae*. The RB group was distinguished by enrichment of *g_norank_f_Anaerovoracaceae*. Collectively, these findings demonstrate that LCB treatment significantly alters the gut microbial composition in mice with rivaroxaban-induced colonic bleeding.

### LCB alters the fecal metabolic profile in mice with rivaroxaban-induced colonic bleeding

To further elucidate the mechanisms underlying the protective effect, we performed untargeted metabolomic analysis of fecal samples using LC-MS. PCA revealed distinct separation of metabolic profiles among the NC, R, and RB groups, indicating differences in their overall fecal metabolic profiles (Figure 3A).

**FIGURE 3:**
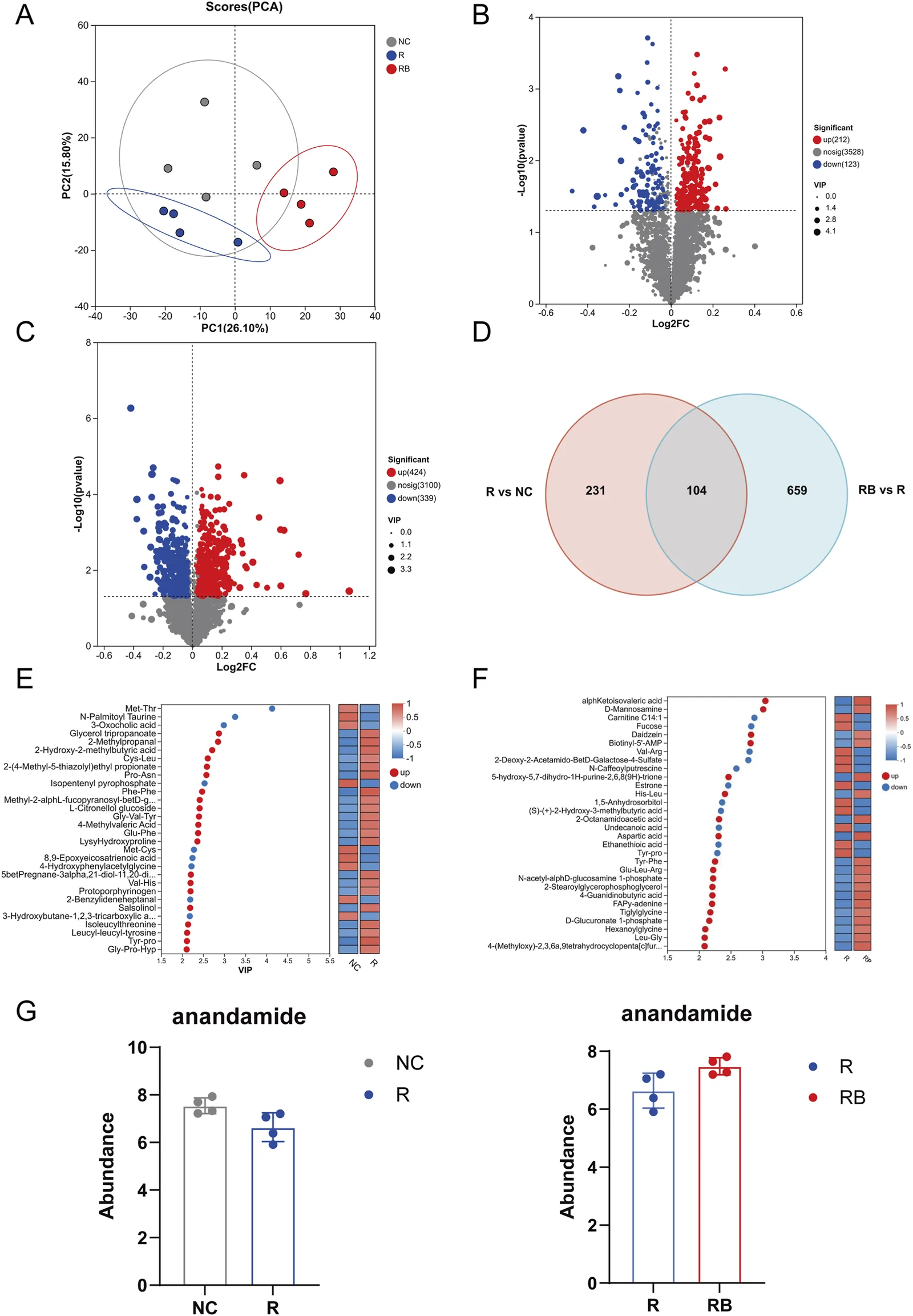
Effect of LCB on the fecal metabolic profile of mice with rivaroxaban-induced colonic bleeding. **(A)** PCA score plot showing separation of metabolic profiles among the NC, R, and RB groups. **(B)** Volcano plot showing candidate metabolite features identified using the exploratory criteria of VIP >1 and nominal P < 0.05 in the NC *versus* R comparison. **(C)** Volcano plot showing candidate metabolite features identified using the exploratory criteria of VIP >1 and nominal P < 0.05 in the R *versus* RB comparison. **(D)** Venn diagram illustrating the numbers of unique and shared candidate metabolite features identified using the exploratory screening criteria in the NC *versus* R and R *versus* RB comparisons. **(E)** Heatmap showing the top 30 candidate metabolite features ranked according to VIP scores derived from the OPLS-DA model for the NC group *versus* R group comparison. **(F)** Heatmap showing the top 30 candidate metabolite features ranked according to VIP scores derived from the OPLS-DA model for the R group *versus* RB group comparison. **(G)** Abundance of the metabolite feature putatively annotated as anandamide. The feature showed a nominal decrease in the R group relative to the NC group (nominal P = 0.0392, FDR-adjusted P = 0.4154) and a nominal increase in the RB group relative to the R group (nominal P = 0.0464, FDR-adjusted P = 0.1904). Neither comparison remained significant after FDR correction. Data are presented as mean ± SD (n = 4 randomly selected mice per group for metabolomics).

The OPLS-DA models used for VIP estimation were evaluated using seven-fold cross-validation and 200-permutation testing. For the NC *versus* R comparison, the model yielded R^2^X = 0.373, R^2^Y = 0.996, and Q^2^ = 0.670. In permutation testing, no permuted model exceeded the observed Q^2^ (0/200; platform-reported P < 0.05), whereas 69 of 200 permutations exceeded the observed R^2^Y (P = 0.345). For the R *versus* RB comparison, the model yielded R^2^X = 0.495, R^2^Y = 0.998, and Q^2^ = 0.865; the corresponding permutation results were 1/200 for R^2^Y and 7/200 for Q^2^ (both platform-reported P < 0.05). Given the small sample size and the non-uniform permutation support across model metrics, VIP-based feature prioritization was interpreted as exploratory.

Using the exploratory screening criteria of VIP >1 and nominal P < 0.05, 212 metabolite features were increased and 123 were decreased in the R group relative to the NC group, whereas 424 features were increased and 339 were decreased in the RB group relative to the R group (Figures 3B,C). After FDR correction, no candidate features in the NC *versus* R comparison remained significant at FDR <0.05, whereas 85 candidate features in the R *versus* RB comparison remained statistically supported after multiple-testing correction. The corresponding nominal and FDR-adjusted P values are provided in Supplementary Tables S1A,B. In total, 104 metabolite features overlapped between the exploratory candidate sets identified in the NC *versus* R and R *versus* RB comparisons and were retained for further exploratory analysis (Figure 3D).

The top 30 candidate metabolite features ranked according to VIP scores derived from the corresponding OPLS-DA models are presented in the heatmaps (Figures 3E,F). Among the exploratory candidate metabolite features, rivaroxaban treatment decreased the levels of metabolites such as Met-Thr, N-palmitoyl taurine, and 3-oxocholic acid, while increasing glycerol tripropanoate, 2-methylpropanal, and 2-hydroxy-2-methylbutyric acid compared to the NC group. In contrast, treatment with LCB lowered levels of carnitine C14:1, fucose, and Val-Arg, and elevated those of α-ketoisovaleric acid, D-mannosamine, daidzein, and biotinyl-5′-AMP relative to the R group.

The metabolite feature putatively annotated as anandamide showed a consistent exploratory pattern across the experimental comparisons. Its abundance was nominally lower in the R group than in the NC group (nominal P = 0.0392, FDR = 0.4154) and nominally higher in the RB group than in the R group (nominal P = 0.0464, FDR = 0.1904) (Figure 3G).

### LCB has a diminished protective effect against rivaroxaban-induced colonic bleeding in antibiotic-treated mice

To assess the contribution of the gut microbiota to the ameliorative effect of LCB, we established an antibiotic-treated mouse model by administering an antibiotic cocktail via oral gavage to deplete the gut microbiota prior to rivaroxaban and probiotic treatment (Figure 4A). To evaluate the effect of antibiotic pretreatment on the gut microbiota, an independent preliminary cohort was analyzed by 16S rRNA gene sequencing. Antibiotic treatment significantly reduced Chao1 richness and Shannon diversity (two-sided Wilcoxon rank-sum tests, both P < 0.001) and altered overall microbial community structure (PERMANOVA: pseudo-F = 8.9057, *R* ^2^ = 0.52679, P = 0.009) (Supplementary Figures S1A-C). Clear changes in relative microbial composition were also observed at the phylum and genus levels. These findings support substantial antibiotic-mediated disruption of the gut microbial community.

**FIGURE 4:**
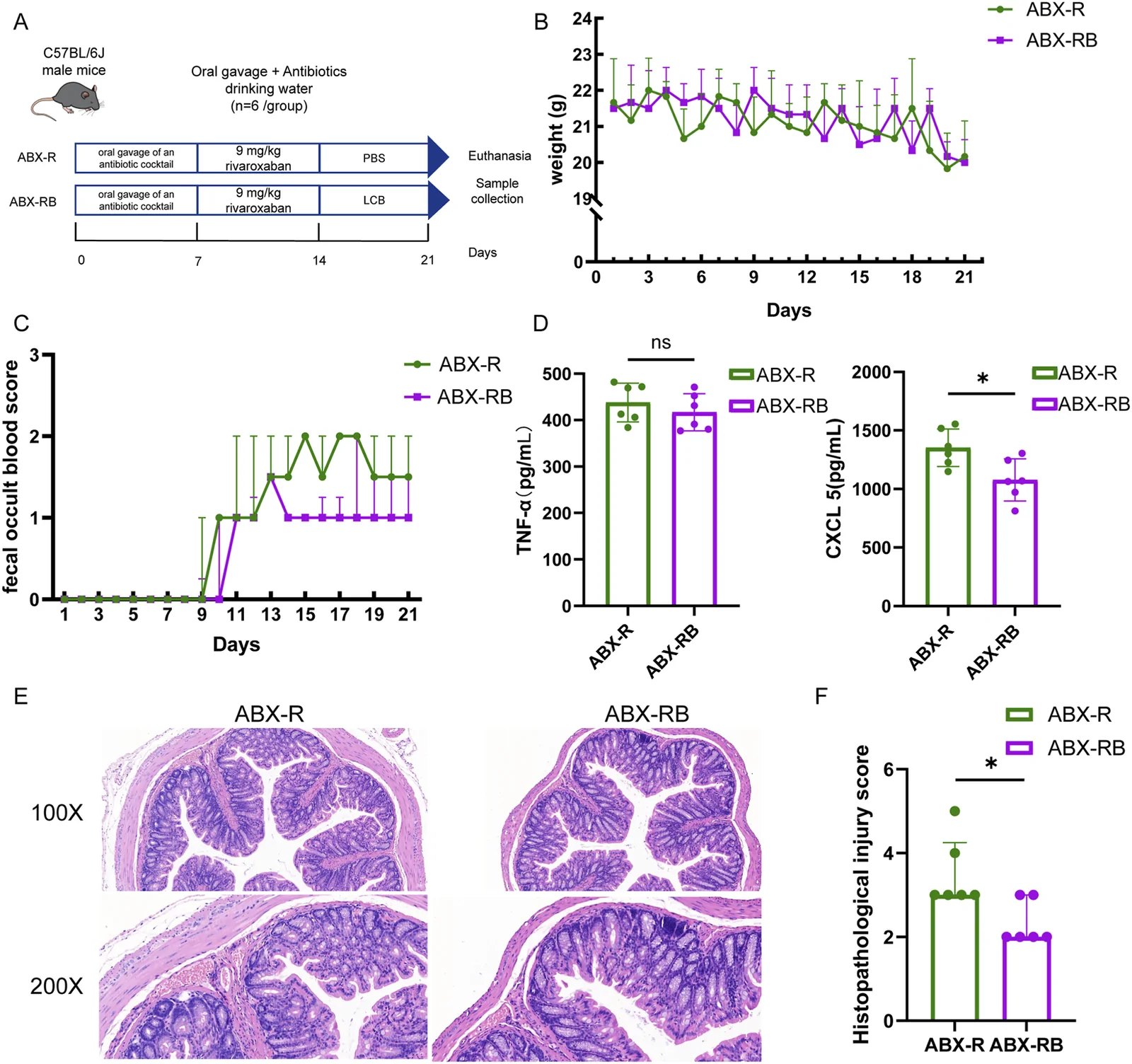
LCB has a diminished protective effect against rivaroxaban-induced colonic bleeding in antibiotic-treated mice. **(A)** Schematic diagram of the experimental protocol for establishing the antibiotic-treated mouse model and subsequent interventions. **(B)** Changes in body weight of mice throughout the experimental period. **(C)** Severity of occult blood in the feces of mice. **(D)** Serum levels of TNF-α and CXCL5. **(E)** Representative H&E-stained colon sections at 100× and 200× total magnification. Scale bars: 50 μm for the 100× images and 25 μm for the 200× images. **(F)** Blinded semiquantitative histopathological injury scores. Continuous data are presented as mean ± SD, whereas fecal occult blood scores and histopathological injury scores are presented as median with interquartile range (n = 6 per group). *P < 0.05.

Two-way repeated-measures ANOVA with Geisser–Greenhouse correction revealed a significant effect of time on body weight [F (6.520, 65.20) = 3.556, P = 0.0033], but no significant group effect [F (1, 10) = 0.4063, P = 0.5382] or group × time interaction [F (6.520, 65.20) = 1.816, P = 0.1040] (Figure 4B). Following rivaroxaban administration, fecal occult blood became detectable in both the ABX-R and ABX-RB groups (Figure 4C). During the LCB intervention period (days 15–21), fecal occult blood scores were numerically lower in the ABX-RB group, but no between-group differences remained significant after Bonferroni adjustment (all adjusted P > 0.05). These findings suggest that the effect of LCB on fecal occult blood was attenuated following antibiotic-mediated microbiota depletion. Serum TNF-α levels were not significantly different between the ABX-R and ABX-RB groups, whereas CXCL5 levels were significantly lower in the ABX-RB group (P < 0.05) (Figure 4D). These findings indicate that LCB retained a partial effect on inflammatory markers following antibiotic-mediated microbiota depletion, although the broader anti-inflammatory response observed in conventional mice was attenuated. Finally, histopathological examination revealed capillary congestion and inflammatory cell infiltration in the ABX-R group. Notably, these pathological changes were only minimally improved in the ABX-RB group (Figure 4E). Blinded histopathological scoring showed that the total injury score was lower in the ABX-RB group than in the ABX-R group (Figure 4F). This result indicates that a partial histopathological improvement remained under antibiotic-treated microbiota-depleted conditions, although the overall ameliorative response was less pronounced than that observed in conventional mice.

### LCB modulates the gut microbiota in antibiotic-treated mice with rivaroxaban-induced colonic bleeding

To further investigate whether the gut microbiota mediates the effects of LCB under antibiotic-treated conditions, we performed 16S rRNA gene sequencing on fecal samples from the ABX-R and ABX-RB groups. Alpha diversity analysis revealed no significant differences in the Shannon or Simpson indices between the ABX-R and ABX-RB groups, as assessed using two-sided Wilcoxon rank-sum tests (Figure 5A). PCoA based on Bray–Curtis dissimilarities demonstrated separation of the gut microbial communities between the two groups, and ANOSIM indicated a significant difference in overall community structure (R = 0.875, P = 0.034) (Figure 5B).

**FIGURE 5:**
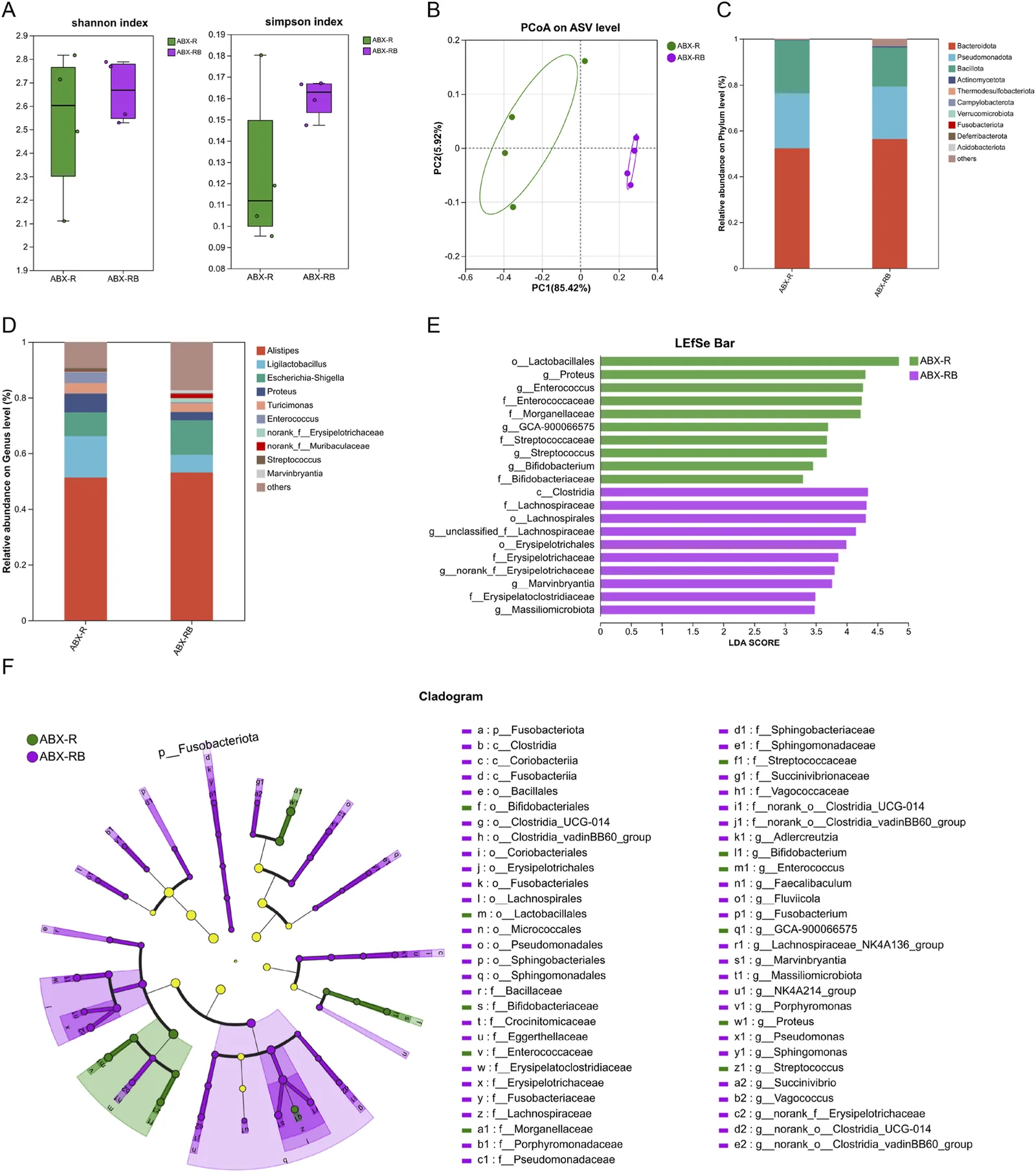
Effect of LCB on the gut microbiota in antibiotic-treated mice with rivaroxaban-induced colonic bleeding. **(A)** Alpha diversity assessed using the Shannon and Simpson indices; between-group comparisons were performed using two-sided Wilcoxon rank-sum tests. **(B)** PCoA based on Bray–Curtis dissimilarities; differences in overall community structure were evaluated using ANOSIM (R = 0.875, P = 0.034). **(C)** Gut microbial composition at the phylum level. **(D)** Gut microbial composition at the genus level. **(E)** LDA score plot showing discriminative taxa between the ABX-R and ABX-RB groups. **(F)** LEfSe cladogram showing the phylogenetic distribution of discriminative taxa. LEfSe features were identified using P < 0.05 and an LDA score >2.0. Data are based on n = 4 randomly selected mice per group for 16S rRNA sequencing.

Analysis of microbial composition at the phylum level showed that Bacteroidota was the dominant phylum in both groups (Figure 5C). At the genus level, LCB treatment resulted in increased relative abundance of Escherichia-Shigella, norank_f_Erysipelotrichaceae, and norank_f_Muribaculaceae compared to the ABX-R group. Conversely, the relative abundance of *Ligilactobacillus*, *Proteus*, and *Enterococcus* was decreased in the ABX-RB group (Figure 5D).

LEfSe was performed to identify specific bacterial taxa affected by probiotic treatment under antibiotic-treated conditions. This analysis revealed that o*_Lactobacillales*, f*_Enterococcaceae*, f*_Morganellaceae*, g*_Proteus*, and g*_Enterococcus* were enriched in the ABX-R group. In contrast, c*_Clostridia*, f*_Lachnospiraceae*, o*_Lachnospirales*, and g*_unclassified_f_Lachnospiraceae* were significantly enriched in the ABX-RB group (Figures 5E,F). The phylogenetic distribution of these differentially abundant taxa is illustrated in the cladogram in the figure. Collectively, these findings demonstrate that LCB treatment modulates the gut microbial composition in antibiotic-treated mice with rivaroxaban-induced colonic bleeding.

### LCB alters the fecal metabolic profile in antibiotic-treated mice with rivaroxaban-induced colonic bleeding

To determine whether LCB-associated metabolic responses persisted following antibiotic-mediated microbiota depletion, we performed untargeted metabolomic analysis of fecal samples from antibiotic-treated mice. PCA revealed a clear separation of metabolic profiles between the ABX-R and ABX-RB groups, indicating distinct differences in their overall fecal metabolic profiles (Figure 6A). The OPLS-DA model used for VIP estimation was evaluated using seven-fold cross-validation and 200-permutation testing. For the ABX-R *versus* ABX-RB comparison, the model yielded R^2^X = 0.414, R^2^Y = 1.000, and Q^2^ = 0.845. In permutation testing, no permuted model exceeded the observed R^2^Y or Q^2^ values (0/200 for both; platform-reported P < 0.05). Nevertheless, because only four mice per group were included, VIP-based feature prioritization was retained as exploratory. Using the same exploratory criteria, 177 metabolite features were increased and 152 were decreased in the ABX-RB group relative to the ABX-R group. Among these candidate features, 71 remained significant after FDR correction (FDR <0.05) (Figure 6B), with the corresponding nominal and FDR-adjusted P values provided in Supplementary Table S1C. The top 30 candidate metabolite features ranked according to VIP scores derived from the OPLS-DA model are presented in the heatmap (Figure 6C). Among the exploratory candidate metabolite features, LCB treatment resulted in increased levels of metabolites such as xanthosine, indoleacetaldehyde, and 4-trimethylammoniobutanoate compared to the ABX-R group. Conversely, levels of pentadecanoic acid, 2-methylpropanal, pentanal, and 2-ethyl-2-hydroxybutyric acid were decreased in the ABX-RB group. The putative anandamide feature also showed a nominal increase in the ABX-RB group compared with the ABX-R group (nominal P = 0.0109, FDR = 0.1465), consistent with the direction observed following LCB treatment in conventional mice (Figure 6D). These findings suggest that certain LCB-associated metabolic responses can be retained following antibiotic-mediated microbiota depletion. In contrast, the overall ameliorative phenotype was attenuated under these conditions, despite residual responses including reduced CXCL5 levels and partial histopathological improvement. Together, these findings indicate that LCB-associated metabolic remodeling is partly retained after microbiota depletion, whereas its overall ameliorative effect appears to involve a broader microbiota-associated metabolic response rather than a single metabolite change.

**FIGURE 6:**
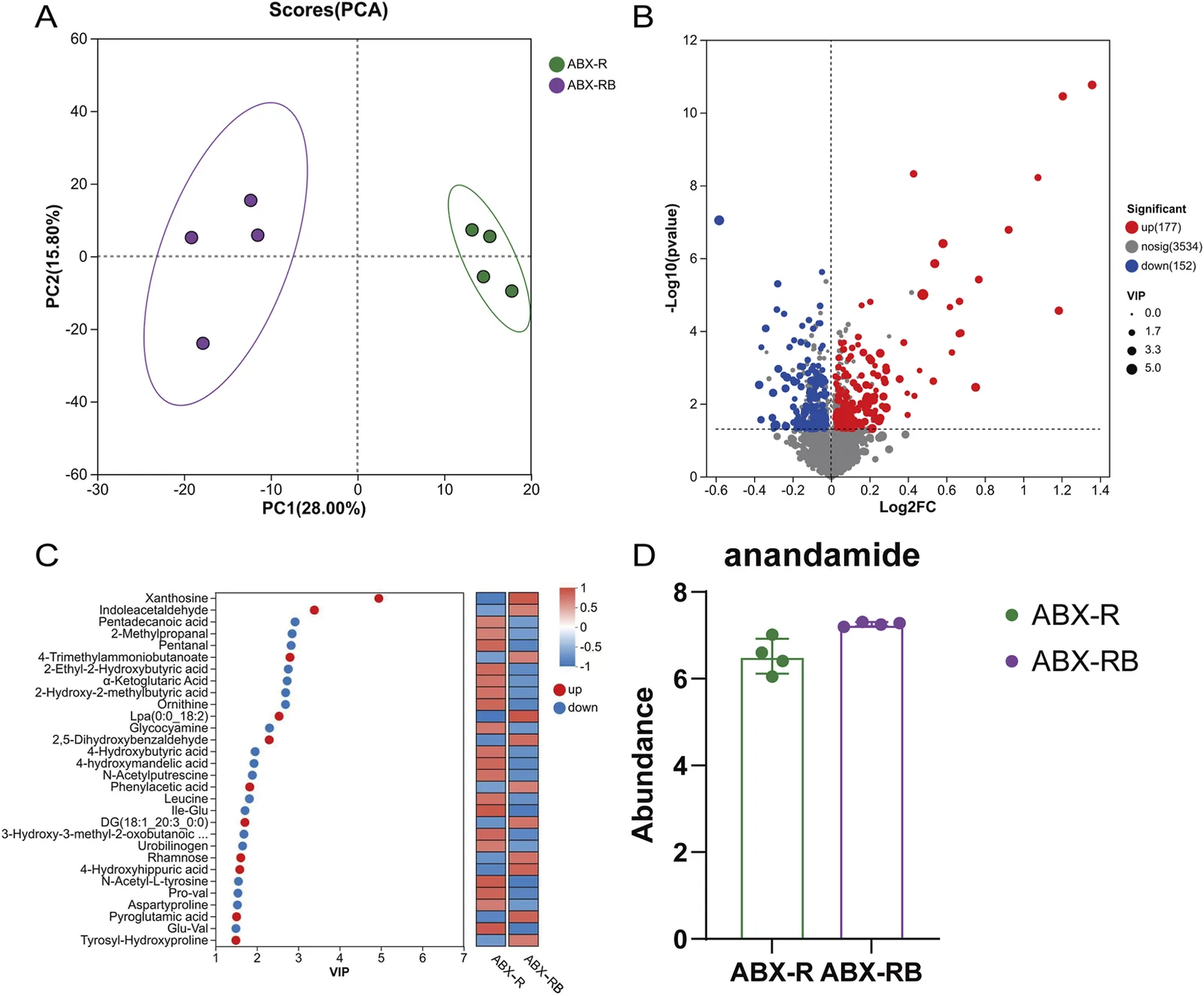
Effect of LCB on the fecal metabolic profile in antibiotic-treated mice with rivaroxaban-induced colonic bleeding. **(A)** PCA score plot showing separation of metabolic profiles between the ABX-R and ABX-RB groups. **(B)** Volcano plot showing candidate metabolite features identified using the exploratory criteria of VIP >1 and nominal P < 0.05 in the ABX-R *versus* ABX-RB comparison. **(C)** Heatmap showing the top 30 candidate metabolite features ranked according to VIP scores derived from the OPLS-DA model for the ABX-R group *versus* ABX-RB group comparison. **(D)** Abundance of the metabolite feature putatively annotated as anandamide in the ABX-R and ABX-RB groups. The feature showed a nominal increase in the ABX-RB group relative to the ABX-R group (nominal P = 0.0109, FDR-adjusted P = 0.1465), but the difference did not remain significant after FDR correction. Data are presented as mean ± SD (n = 4 randomly selected mice per group for metabolomics).

### Analysis of associations between gut microbiota and the metabolite feature putatively annotated as anandamide

Given its consistent directional pattern across the experimental comparisons and its biological plausibility, the putative anandamide feature was selected for exploratory correlation analysis with the gut microbiota (Figure 7). Spearman’s rank correlation analysis was performed using paired microbiome and metabolomic data from 20 mice across the five experimental groups (n = 4 per group). Using the predefined exploratory criteria of |ρ| > 0.4 and nominal P < 0.05, eight associations were identified, comprising seven positive and one negative correlation (Figure 7). Three Lachnospiraceae-associated taxa showed nominal positive correlations with the putative anandamide feature: Lachnospiraceae_FCS020_group (ρ = 0.5684, nominal P = 0.0089), Lachnospiraceae_NK4A136_group (ρ = 0.4962, nominal P = 0.0261), and norank_f__Lachnospiraceae (ρ = 0.4642, nominal P = 0.0392). However, none of the eight displayed associations remained significant after Benjamini–Hochberg correction (all BH-adjusted P > 0.05). The complete recalculated correlation results are provided in Supplementary Table S2. Accordingly, these associations were interpreted as exploratory and hypothesis-generating rather than confirmatory.

**FIGURE 7:**
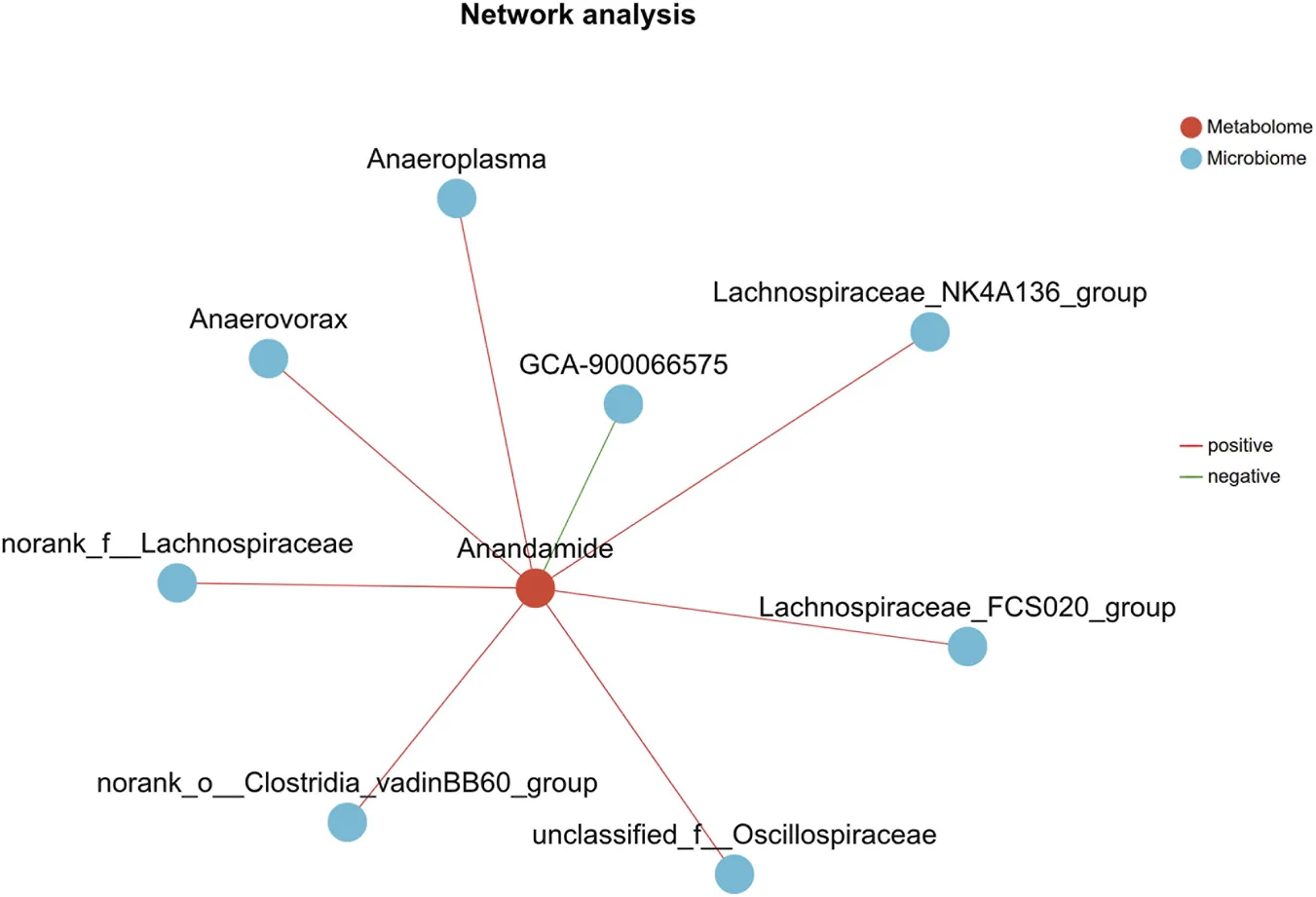
Exploratory correlation network between gut microbial genera and the metabolite feature putatively annotated as anandamide. Spearman’s rank correlations were calculated using paired microbiome and metabolomic measurements from 20 mice across the five experimental groups (n = 4 per group). Network edges represent associations meeting the predefined exploratory criteria of |ρ| > 0.4 and nominal P < 0.05. Red and green edges indicate positive and negative correlations, respectively. Eight nominal associations were identified. Benjamini–Hochberg correction was additionally applied across all tested correlations, and none of the displayed associations remained significant after multiple-testing correction. Recalculated correlation coefficients, nominal P values, and BH-adjusted P values are provided in Supplementary Table S2.

## Discussion

Rivaroxaban, a widely used DOAC, carries a significant risk of gastrointestinal bleeding, which has become a major clinical concern (Patel et al., 2011; Ingason et al., 2021). Increasing attention is focused on the interaction between gut microbiota and gastrointestinal bleeding. Several studies have shown that supplementation with specific probiotic strains, such as *Lactobacillus plantarum* Lpf-01 and *Bifidobacterium longum* K5, can ameliorate nonsteroidal anti-inflammatory drug-induced or pathogen-associated gastrointestinal bleeding by enhancing mucosal barrier function, modulating immune responses, and suppressing inflammation (Shan et al., 2024; Zhou et al., 2024; Zou et al., 2024). Probiotic interventions have shown beneficial effects in several models of drug-associated gastrointestinal injury, mainly through modulation of the intestinal microbiota, enhancement of barrier function, and attenuation of inflammatory responses. These observations provide a rationale for exploring microbiota-targeted approaches to ameliorate rivaroxaban-associated gastrointestinal injury.

Clinical data confirm that rivaroxaban is associated with a higher risk of gastrointestinal bleeding compared to other DOACs, and cases of colonic bleeding have been documented (Hirano et al., 2018; Logvinsky et al., 2025). Beyond its systemic anticoagulant effects, rivaroxaban may impair mucosal healing by inhibiting factor Xa. Its active form, excreted into the intestine, may directly irritate vulnerable mucosa, further increasing bleeding risk (Desai et al., 2013; Cheung and Leung, 2017; Saviano et al., 2022). In our study, rivaroxaban administration induced persistent fecal occult blood, capillary congestion, and inflammatory cell infiltration in colon tissues. It is well established that injury to the gastrointestinal mucosa can upregulate the local expression of inflammatory cytokines (Fan et al., 2021). TNF-α, drives endothelial damage and vascular permeability (Groten et al., 2025; Jia et al., 2025), while CXCL5 promotes neutrophil recruitment (Vieira et al., 2009; Disteldorf et al., 2015), matching histological evidence of inflammatory infiltration.

This inflammatory cascade, characterized by TNF-α and CXCL5 overproduction, disrupts barrier integrity and exacerbates bleeding. Notably, this study provides the first evidence that intervention with LCB effectively suppresses TNF-α and CXCL5, interrupting this cascade and mitigating colonic injury. These findings align with recent reports that probiotic strains such as *Lactobacillus* plantarum LPF-01 and *Lactobacillus sakei* LPG-9 alleviate gastric injury induced by nonsteroidal anti-inflammatory drugs by enhancing barrier function and modulating signaling pathways (Shan et al., 2024; Zhou et al., 2024). Collectively, our results suggest that probiotic modulation of the gut microbiota represents a promising strategy for treating drug-induced gastrointestinal injury.

Analysis of the gut microbiota provided mechanistic insight into the protective effects observed. Rivaroxaban administration disrupted intestinal microecological balance, enriching pro-inflammatory phyla such as Bacteroidota, consistent with prior reports of rivaroxaban induced microbial alteration (Liu et al., 2025). At the genus level, rivaroxaban increased bacteria linked to mucosal polysaccharide metabolism (e.g., *norank_f_Muribaculaceae*) while significantly reducing beneficial taxa such as Lachnospiraceae_NK4A136_group. Clinical data similarly show reduced Lachnospiraceae abundance in patients with atrial fibrillation receiving oral anticoagulants (Li et al., 2023). Because Lachnospiraceae are major producers of short-chain fatty acids (SCFAs), their depletion compromises epithelial barrier integrity and promotes inflammation (Zheng et al., 2020; Liu et al., 2026).

Conversely, rivaroxaban enriched Prevotellaceae_UCG-001, a genus with recognized pro-inflammatory potential (Larsen, 2017; Iljazovic et al., 2021). *Prevotella* species secrete sulfatases that degrade mucin, weakening the intestinal mucus barrier and permitting Gram-negative bacteria to translocate and contact epithelial cells, activating the NLRP3 inflammasome and promoting inflammatory responses (Elinav et al., 2011). Furthermore, increased Prevotellaceae_UCG-001 abundance has been linked to elevated pro-inflammatory cytokines and reduced expression of tight junction proteins, further implicating this genus in intestinal inflammation (Hu et al., 2025). Thus, rivaroxaban-induced imbalance, characterized by loss of SCFA-producing Lachnospiraceae and expansion of pro-inflammatory Prevotellaceae, likely exacerbates mucosal injury and bleeding risk. Importantly, treatment with LCB effectively reversed these microbial shifts, restoring beneficial taxa and suppressing harmful ones. These findings suggest that probiotic modulation of gut microbiota may represent a promising strategy to mitigate DOAC-associated gastrointestinal bleeding.

Metabolomic analysis further elucidated the connection between changes in the gut microbiota and the observed protective effects. Untargeted metabolomic analysis identified a feature putatively annotated as anandamide that showed a nominal decrease following rivaroxaban administration and a nominal increase following LCB treatment. Although these differences did not remain significant after FDR correction, the consistency of their directional pattern across the experimental comparisons, together with the reported biological functions of anandamide, motivated exploratory follow-up of this feature. Anandamide, an endocannabinoid, plays a pivotal role not only in pain and inflammation regulation but also in protecting the gastrointestinal mucosa and modulating blood flow (Sharkey and Tsang, 2025; Sharma et al., 2025). It produces its anti-inflammatory effects by activating cannabinoid receptor type 2 expressed on immune cells, thereby inhibiting the release of pro-inflammatory cytokines and promoting the production of anti-inflammatory cytokines (Varsha et al., 2022). Notably, in a previous study that employed a mouse model of inflammation, anandamide treatment reversed gut microbiota dysbiosis induced by staphylococcal enterotoxin B-mediated acute respiratory distress syndrome and increased the relative abundance of butyrate-producing Lachnospiraceae and Clostridia (Sultan et al., 2021). In the exploratory pooled correlation analysis, several Lachnospiraceae genera showed nominal positive correlations with the putative anandamide feature. In the recalculated exploratory analysis based on 20 paired samples, three Lachnospiraceae-associated taxa showed nominal positive correlations with the putative anandamide feature. However, none of these associations remained significant after Benjamini–Hochberg correction. Moreover, because the analysis pooled mice across five experimental groups, the observed correlations may partly reflect treatment-related between-group differences. These findings should therefore be regarded as hypothesis-generating and do not establish a causal relationship between Lachnospiraceae and anandamide metabolism.

Importantly, the effects of LCB were not uniformly abolished following antibiotic-mediated microbiota depletion. Although the reduction in fecal occult blood scores was not statistically significant and TNF-α remained unchanged, CXCL5 was significantly reduced and partial histopathological improvement persisted. Thus, microbiota depletion attenuated the integrated ameliorative response to LCB rather than completely eliminating all LCB-associated effects. This dissociation suggests that anandamide elevation alone is insufficient to account for the protective effect of LCB. It remains possible that anandamide acts in combination with other microbiota-dependent metabolites, bacterial functions, or host responses; however, this possibility cannot be resolved by the present data. Targeted LC-MS/MS analysis using an authentic standard and an isotope-labelled internal standard will be required to independently confirm the identity and absolute concentration of anandamide. In addition, functional studies involving exogenous anandamide administration, pharmacological modulation of anandamide degradation, and/or cannabinoid receptor blockade will be necessary to determine whether anandamide contributes causally to the protective phenotype. Accordingly, the present anandamide findings should be interpreted as exploratory and hypothesis-generating.

The antibiotic-depletion experiment was important for distinguishing residual biological responses from the integrated gastrointestinal protective effect of LCB. Although LCB retained some capacity to modify microbial and metabolic features after antibiotic pretreatment, its overall ability to ameliorate rivaroxaban-associated colonic injury was markedly reduced. These findings suggest that interactions with the resident microbial ecosystem contribute substantially to the full ameliorative effect of LCB.

Nevertheless, LCB continued to alter microbial composition and fecal metabolic profiles under antibiotic-treated conditions, indicating that certain microbiological and metabolic responses were retained. Taken together, these findings support a model in which the full protective effect of LCB is enabled by coordinated interactions among the administered probiotic strains, the residual or resident microbiota, microbial and host metabolites, and host inflammatory pathways, rather than by a single bacterial taxon or metabolite.

Several limitations should be acknowledged. First, this study was conducted in a murine model, and the findings therefore require validation in clinical populations. Second, LCB was administered after rivaroxaban exposure and the onset of fecal occult bleeding, while rivaroxaban was not continued during the subsequent intervention period; thus, the present design does not address prophylactic efficacy or the effects of LCB during ongoing anticoagulant treatment. Third, although the rivaroxaban-treated groups received identical DMSO exposure, the NC group received PBS without DMSO, and a minor contribution of the low DMSO concentration to comparisons with the NC group cannot be completely excluded. In addition, antibiotic-mediated microbiota disruption was evaluated in an independent preliminary cohort rather than longitudinally in the animals used in Experiment 2, and absolute bacterial load was not quantified by qPCR, bacterial culture, flow cytometry, or another quantitative method. Therefore, these data support substantial microbiota disruption but do not establish complete or quantitatively verified bacterial depletion. Furthermore, the untargeted metabolomic analyses were performed in only four mice per group. Nominal P values were derived from two-sided unpaired Student’s t-tests without formal feature-wise assessment of normality or homogeneity of variance, and the small sample size also limits the stability of the OPLS-DA models despite seven-fold cross-validation and 200-permutation testing. Accordingly, metabolite features meeting VIP >1 and nominal P < 0.05 but not the FDR threshold were treated as exploratory. In particular, the putative anandamide feature did not remain significant after FDR correction and should therefore be regarded as hypothesis-generating pending targeted validation in larger independent cohorts. The microbiota–anandamide correlation analysis was exploratory and was based on pooled data across five experimental groups. Although Benjamini–Hochberg correction was applied across all tested correlations, none of the displayed associations remained significant after multiple-testing correction. Therefore, these nominal associations require confirmation in larger independent datasets.

## Conclusion

In conclusion, LCB attenuated rivaroxaban-associated colonic bleeding and injury in mice. The marked reduction in its overall ameliorative effects following antibiotic-mediated microbiota depletion supports a substantial microbiota-dependent component. LCB treatment was also accompanied by alterations in a metabolite feature putatively annotated as anandamide and nominal exploratory associations between this feature and three Lachnospiraceae-associated taxa; however, these associations did not remain significant after Benjamini–Hochberg correction. However, the persistence of the putative anandamide increase under antibiotic-treated conditions without consistent restoration of the protective phenotype indicates that anandamide elevation alone is insufficient. Its causal contribution remains unproven and requires targeted quantitative and functional validation.

## Data Availability

The raw 16S rRNA gene sequencing data generated in this study have been deposited in the NCBI Sequence Read Archive (SRA) under BioProject accession number PRJNA1507308. The raw untargeted metabolomics data generated in this study have been deposited in the MetaboLights repository under accession number MTBLS15531.
