# Supplementary material for: Live combined bifidobacterium attenuates rivaroxaban-associated colonic bleeding in mice: evidence for a substantial gut microbiota-dependent component
Source: Front Pharmacol. 2026 Oct 21;17:1846463. doi: 10.3389/fphar.2026.1846463 (PMC13730847; doi:10.3389/fphar.2026.1846463)
Supplement: Supplementary file 1 [file Image1.pdf]

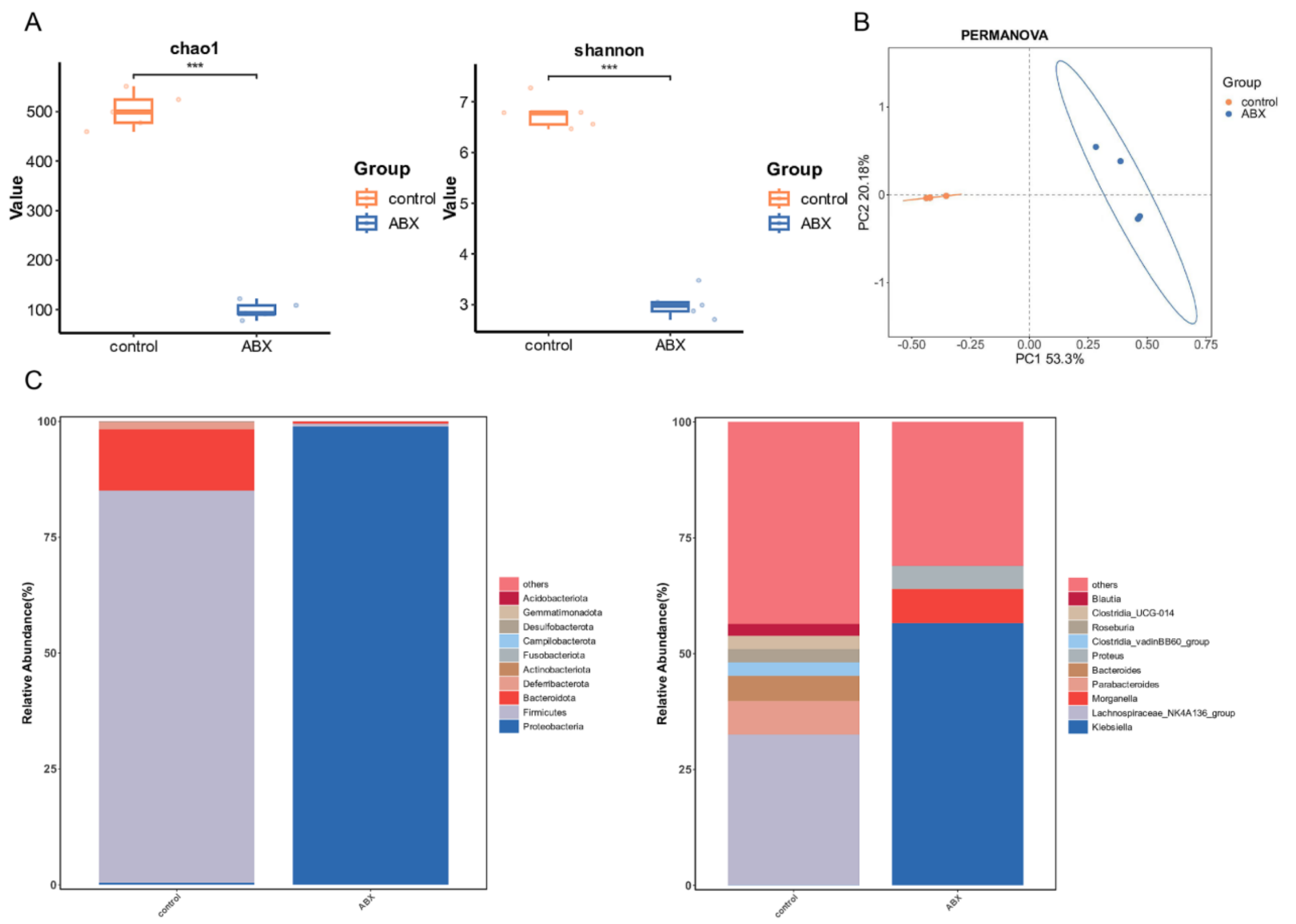

Supplementary Figure S1. Assessment of antibiotic-induced alterations in the gut microbial community in an independent preliminary cohort. (A) Alpha diversity of the gut microbiota in the control and antibiotic-treated (ABX) groups, assessed using the Chao1 richness and Shannon diversity indices. Between-group comparisons were performed using two-sided Wilcoxon rank-sum tests. Both indices were significantly reduced following antibiotic treatment. (B) Principal coordinate analysis (PCoA) based on Bray–Curtis dissimilarities showing separation of the gut microbial communities between the control and ABX groups. Differences in overall community structure were assessed using PERMANOVA (pseudo-F = 8.9057, R^2 = 0.52679, P = 0.009). (C) Relative abundance profiles of the dominant bacterial taxa at the phylum level (left) and genus level (right) in the control and ABX groups. Fecal samples were collected after 7 days of antibiotic treatment and before subsequent rivaroxaban or LCB administration. Data were obtained from n = 5 mice per group. *P < 0.001.
